# Amelioration of endotoxin-induced uveitis treated with an IκB kinase β inhibitor in rats

**Published:** 2012-10-20

**Authors:** Anton Lennikov, Nobuyoshi Kitaichi, Kousuke Noda, Ryo Ando, Zhenyu Dong, Junichi Fukuhara, Satoshi Kinoshita, Kenichi Namba, Miho Mizutani, Tomoyuki Fujikawa, Akiko Itai, Shigeaki Ohno, Susumu Ishida

**Affiliations:** 1 Laboratory of Ocular Cell Biology and Visual Science, Department of Ophthalmology, Hokkaido University Graduate School of Medicine, Sapporo, Japan; 2 Department of Ocular Inflammation and Immunology, Hokkaido University Graduate School of Medicine, Hokkaido University Graduate School of Medicine, Sapporo, Japan; 3 Department of Ophthalmology, Health Sciences University of Hokkaido, Sapporo, Japan; 4 Institute of Medicinal Molecular Design (IMMD) Inc, Tokyo, Japan

## Abstract

**Purpose:**

Endotoxin-induced uveitis (EIU) is an animal model for acute ocular inflammation. Several substances play major roles in the development of inflammatory changes in EIU, including tumor necrosis factor-α (TNF-α), interleukin (IL)-1β, and IL-6. These inflammatory cytokines trigger the degradation of IκB by activating IκB kinases (IKKs). Released nuclear factor kappaB (NFκB) subsequently translocates to the nucleus, where NFκB expresses its proinflammatory function. IMD-0354, N-(3,5-Bis-trifluoromethylphenyl)-5-chloro-2-hydroxybenzamide, selectively inhibits IKKβ, particularly when induced by proinflammatory cytokines, such as TNF-α and IL-1β. In the present study, we examined whether IKKβ inhibition has therapeutic effects on EIU by using IMD-0354 and its prodrug IMD-1041.

**Methods:**

Six-week-old male Lewis rats were used. EIU was induced with subcutaneous injections of 200 μg of lipopolysaccharide (LPS) from *Escherichia coli* that had been diluted in 0.1 ml of phosphate-buffered saline. IMD-0354 was administered intraperitoneally at 30, 10, 3, or 0 mg/kg, suspended in 1.0 ml of 0.5% carboxymethyl cellulose sodium. The prodrug IMD-1041 (100 mg/kg) was also administered orally. The rats were euthanized 24 h after LPS injection, and EIU severity was evaluated histologically. The number of infiltrating cells and the protein, TNF-α, and monocyte chemoattractant protein-1 (MCP-1) concentrations in the aqueous humor were determined. TNF-α and MCP-1 concentrations were quantified with enzyme-linked immunosorbent assay. Eye sections were also stained with anti-NFκB and phosphorylated I-κBα antibodies.

**Results:**

The number of infiltrating cells in aqueous humor was 53.6±9.8×10^5^, 72.5±17.0×10^5^, 127.25±32.0×10^5^, and 132.0±25.0×10^5^ cells/ml in rats treated with 30, 10, 3, or 0 mg/kg of IMD-0354, respectively. The total protein concentrations of aqueous humor were 92.6±3.1 mg/ml, 101.5±6.8 mg/ml, 112.6±1.9 mg/ml, and 117.33±1.8 mg/ml in rats treated with 30, 10, 3, and 0 mg/kg of IMD-0354, respectively. Infiltrating cells and protein concentrations were significantly decreased by treatment with IMD-0354 (p<0.01). IMD-0354 treatment significantly reduced the concentration of TNF-α (p<0.05) and MCP-1 (p<0.01) in aqueous humor. The number of NFκB positive nuclei was reduced when treated with IMD-0354. Furthermore, IMD-0354-treated EIU rats showed only background levels of phosphorylated I-κBα; however, it was strongly expressed in the iris-ciliary body cell cytoplasm of the IMD-0354 untreated EIU rats. Oral administration of IMD-1041 also decreased the cell number (p<0.01) and protein concentration (p<0.05) of aqueous humor in EIU.

**Conclusions:**

Acute uveitis was ameliorated by inhibition of IKKβ in rats. IMD-0354 and its prodrug IMD-1041 seem to be promising candidates for treating intraocular inflammation/uveitis.

## Introduction

Endotoxin-induced uveitis (EIU) is an animal model of acute anterior segment intraocular inflammation induced by injection of endotoxin, the lipopolysaccharide (LPS) component of the Gram-negative bacterial cell wall [1]. Cellular infiltration and protein leakage into the anterior chamber of the eye reach a maximum at 24 h after LPS injection [2]. Elevated expression of cytokines and chemokines such as tumor necrosis factor (TNF)-α, interleukin (IL)-6, monocyte chemoattractant protein (MCP)-1, and macrophage inflammatory protein (MIP)-2 have been observed concomitant with maximum EIU [2,3]. Other inflammatory mediators, such as nitric oxide [4] and prostaglandin [5], are also involved in the pathogenesis of EIU. The production and release of inflammatory cytokines by LPS depend on inducible gene expression, mediated by the activation of transcription factors. Nuclear factor (NF) κB, one of the most ubiquitous transcription factors, has been suggested to play a key role in these reactions [6]. NFκB exists in the cytoplasm in an inactive form, associated with regulatory proteins called inhibitors of κB (IκB). Phosphorylation of IκB, an important step in NFκB activation, is mediated by an IκB kinase (IKK). The IKK complex consists of at least three subunits, including the kinases IKK-α and IKK-β (also called IKK-1 and IKK-2, respectively) [7] and the regulatory subunit IKK-γ [8]. An inducible form of IKK, known as IKKi, was recently identified in endotoxin-stimulated immune cells [9]. IKK activation initiates IκBα phosphorylation at specific NH_2_-terminal serine residues. Phosphorylated IκBα is then ubiquitinated, which targets it for degradation by the 26S proteasome [10], thus releasing NFκB dimers from the cytoplasmic NFκB–IκB complex and allowing them to translocate to the nucleus. NFκB then binds to κB-enhancer elements of target genes, inducing transcription of proinflammatory genes. Proinflammatory cytokines, such as interleukin-1β (IL-1β) and tumor necrosis factor-α (TNF-α), are regulated by NFκB activation and are known to be the stimuli that activate IκB kinase. Since NFκB is the main factor in the positive feedback loop of inflammation, inhibiting its activation may be an effective therapy for intraocular inflammation.

IMD-0354, IUPAC name N-(3,5-Bis-trifluoromethylphenyl)-5-chloro-2-hydroxybenzamide, was originally designed to competitively interrupt the access of ATP to its docking site on IKKβ, resulting in suppressing the activity of the IKK complex [10]. IMD-0354, a low-molecular-weight compound, has inhibited allergic inflammation in an acute mouse model of asthma [11] and bleomycin-induced lung fibrosis in mice [12]. IMD-0354 selectively inhibits IKKβ, particularly when it is induced by proinflammatory cytokines, such as TNF-α and IL-1β [11-13]. Previous reports showed that IMD-0354 was effective in acute and subacute inflammatory diseases such as myocardial ischemia/reperfusion injury [13] and insulin resistance [14]. These reports also demonstrated the safety of IMD-0354 in vitro and in vivo [11-13].

IMD-1041 is a prodrug of IMD-0354, and oral administration of IMD-1041 has prevented kidney injury in rats [15]. These findings indicate that inhibiting IKKβ with IMD-0354/IMD-1041 may be suitable for clinical treatment of intraocular inflammation. The purpose of this study was to examine the effect of IκB phosphorylation inhibitors in a rat EIU model.

## Methods

### Animals and reagents

IMD-0354, *N*-(3,5-Bis-trifluoromethyl-phenyl)-5-chloro-2-hydroxy-benzamide, and its prodrug IMD-1041 were kindly given by the Institute of Medical Molecular Design (IMMD, Tokyo, Japan). They were dissolved in 0.5% carboxymethylcellulose (CMC) vehicle (Sigma, Tokyo, Japan) immediately before use. Drug-free vehicle (0.5% CMC solution) was used as a control.

Eight-week-old male Lewis rats (180–220 g) were used. All procedures involving animals were performed in accordance with the ARVO resolution on the use of animals in research. EIU was induced with subcutaneous injection with 200 μg LPS from *Escherichia coli* (Sigma-Aldrich, St. Louis, MO) that had been diluted in 200 μl phosphate-buffered saline (PBS; NaCl 8 g, (Na_2_HPO_4_)12H_2_0 2.9 g, KCl 0.2 g, KH_2_PO_4_ 0.2 g, in H_2_O 1 l; pH 7.4). At the same time, the rats were injected intraperitoneally with 30, 10, or 3 mg/kg of IMD-0354, diluted in 500 μl of 0.5% CMC. Control EIU rats were intraperitoneally administered 500 μl of CMC alone (no IMD-0354). Naïve rats were used as controls. All experiments were performed in triplicate with five animals in each group. Unless stated otherwise, all section slide-imaging and evaluation were performed with a BZ-9000 fluorescence microscope (Keyence, Osaka, Japan) and software bundled with the apparatus.

### Histopathological evaluation

Rats were euthanized 24 h after LPS administration by single intravenous injection of sodium pentobarbital (100 mg/kg, Sigma, Tokyo, Japan). The eyes were enucleated immediately and stored in 4% paraformaldehyde in 0.1 M PBS for 24 h, and were then embedded in paraffin. Sagittal sections (5 μm thick) were cut through the optic nerve head and stained with hematoxylin and eosin. The number of infiltrating cells was counted in the two iris-ciliary body (ICB) areas of each slide as well as near the retinas in the posterior area of the eyes, and the results were averaged.

### Number of infiltrating cells and concentrations of protein, tumor necrosis factor-α, and monocyte chemoattractant protein-1 in aqueous humor

At 24 h after LPS injection, rats were euthanized, and the aqueous humor was collected immediately from both eyes with an anterior chamber puncture (15–20 μl/rat) using a 30-gauge needle (Terumo, Tokyo, Japan), under Olympus SZ61 microscope (Olympus, Tokyo, Japan) under a surgical microscope. For cell counting, the aqueous humor sample was suspended in an equal amount of Türk stain solution (Merck, Darmstadt, Germany), and the cells were counted with a hemocytometer under Olympus CKX31 microscope (Olympus). The number of cells was manually counted in each field, and the number of cells per microliter was obtained by averaging the results of four fields from each sample. The total protein concentration in the aqueous humor samples was measured with a bicinchoninic acid protein assay kit (Pierce, Rockford, IL). The aqueous humor samples were stored in ice water until testing, and cell counts and total protein concentrations were measured on the day of sample collection.

The levels of TNF-α and MCP-1 in the aqueous humor were assessed with commercially available ELISA (ELISA) kits: Rat TNF-α ELISA and Rat MCP-1 ELISA according to the manufacturer’s instructions (Thermo Fisher Scientific, Waltham, MA). The detection limits of the ELISA kits were 15 pg/µl and 5 pg/ml for TNF-α and MCP-1, respectively. The ELISA assay was performed in triplicate, with five or more wells involved in each experiment.

### Immunohistochemical studies for nuclear factor kappa B

At 3 h after LPS injection, the rats were euthanized by intravenous injection of sodium pentobarbital (100 mg/kg, Sigma), and the eyes were fixed with an intracardiac perfusion of 4% paraformaldehyde in 0.1 M PBS. The eyes were then enucleated, immersed in the same fixative for 12 h, and then embedded in paraffin. Next, 5-μm sagittal sections were cut near the optic nerve head. Sections were dewaxed with xylene and rehydrated with ethanol. Antigen retrieval was performed by heating sections in a microwave oven in citrate buffer. To evaluate the NFκB-positive cells in the ICB cells, sections were applied with a 1:50 dilution of NFκB P65 antibody (Santa Cruz Biotechnology, Santa Cruz, CA) for 12 h, washed with PBS, and then applied with 1:1000 dilution of secondary goat–anti-rabbit antibody dye conjugate (Invitrogen, Carlsbad, CA), which gives red fluorescence. Since NFκB is widely present in intercellular tissue, to detect its expression in cell nuclei, we stained the section slides with 1:1000 dilution of YO-PRO-1 (Invitrogen), which produces a green signal from the cell nuclei. YO-PRO-1 is a sensitive detector of membrane permeabilization, and a normally cell-impermeant, monomeric, cyanine dye with a strong binding affinity to nucleic acids. NFκB positive (yellow) nuclei were quantified in merged images of the ICB area of the eyes. The results for the two areas were averaged for each sample and in each group. Slide-imaging and evaluation were performed with a FluoView 1000 confocal microscope (Olympus, Tokyo, Japan) and software bundled with the apparatus.

### Immunohistochemical studies for phosphorylated I-κBα

Rat eye fixation and harvesting as well as histological section preparation were identical to the NFκB immunohistochemical staining described above. To evaluate phosphorylated I-κBα expression in the ICB cell cytoplasm, sections were applied with a 1:50 dilution of phosphorylated I-κBα antibody (Cell Signaling Technology, Danvers, MA) for 12 h, washed with PBS, and then applied with 1:1000 dilution of secondary goat–anti-mouse antibody dye conjugate (Invitrogen), which gives green fluorescence. To detect cell nuclei, we stained the section slides with 1:1000 dilution of 4',6-diamidino-2-phenylindole (DAPI; Invitrogen), which produces a blue signal. Sections from EIU rats without phosphorylated I-κBα antibody applied were used as a negative control. The staining protocol was based on recent reports on immunohistochemical detection and evaluation of phosphorylated I-κBα [16].

### Oral administration of IMD-1041

IMD-0354 is available only in injection form; however, the prodrug, IMD-1041 can be administered orally. We performed an additional experiment to examine the effect of oral prodrug IMD-1041 administration on EIU in rats. Eight-week-old male Lewis rats (180–220 g) were orally treated with 100 mg/kg of IMD-1041 dissolved in 1 ml of 0.5% CMC vehicle (Sigma) immediately before use. Drug-free vehicle (0.5% CMC solution) was used as a control. Disposable feeding needles for oral administration were purchased from Fuchigami Inc. (Osaka, Japan). EIU was induced with subcutaneous injection with 200 μg LPS from *E. coli* (Sigma-Aldrich). Control EIU rats were orally administered 500 μl of CMC alone. All experiments were performed in triplicate with four animals in each group. The protein concentration and the number of infiltration cells of the aqueous humor samples were then evaluated.

### Statistical analysis

All values are expressed as mean±standard error of mean (SEM) from the respective groups of experimental or control data. Statistical significance was evaluated with the unpaired Student *t* test. P values less than 0.05 are considered significant.

## Results

### Histopathological findings in eyes of endotoxin-induced uveitis rats

**Figure 1:**
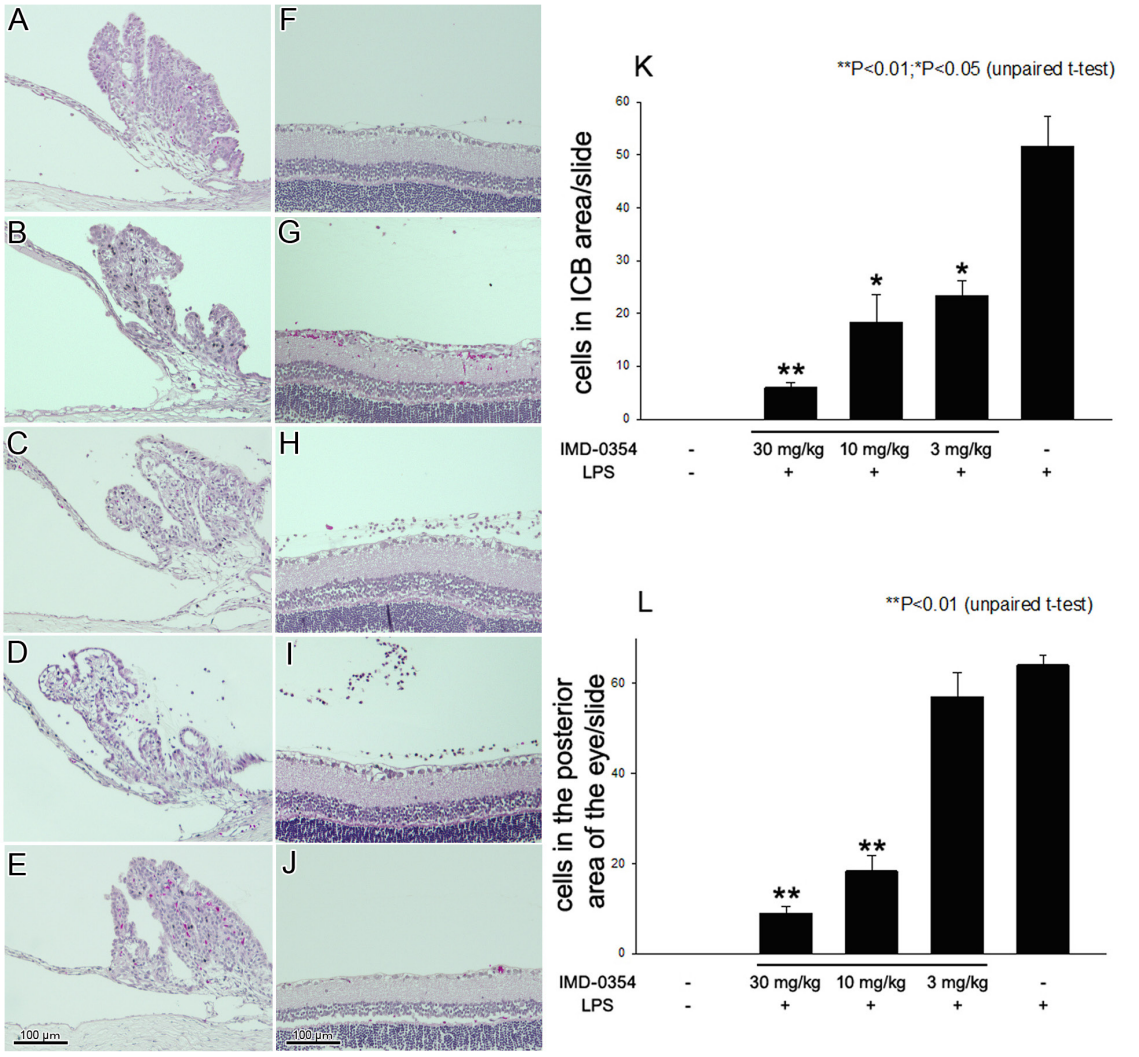
Histological changes in the iris-ciliary body, vitreous cavity, and retina 24 h after lipopolysaccharide injection. Photographs on the left side show the iris-ciliary body (ICB) region, and those on the right side show the vitreous and retina in rats. Control animals **E**, **J:** were not injected with lipopolysaccharide (LPS); no inflammation was observed. Severe inflammatory cell infiltration was observed in endotoxin-induced uveitis (EIU) rats **D**: **I:** In the group of EIU rats treated with IMD-0354 (30 mg/kg; **A**, **F**: 10 mg/kg; **B**, **G:** reductions in cell infiltration were observed compared to untreated EIU rats. No noticeable reduction in cell infiltration was observed in EIU rats treated with IMD-0354 (3 mg/kg: **C**: **H:** Infiltrating cells in the ICB of sections **K**: and in the posterior part of the eye (l) were counted and averaged. Data are shown as mean±standard error of mean (SEM; n=8). *p<0.05, **p<0.01, significantly different from the LPS group.

Only a few inflammatory cells infiltrated in the eyes of rats treated with 30 mg/kg (Figure 1A,F) and 10 mg/kg (Figure 1B,G) of IMD-0354. There was no visible reduction in the number of infiltrating cells in the eyes of rats treated with IMD-0354 (3 mg/kg; Figure 1C,H). Representative histological changes in the eyes of LPS-injected EIU animals that were untreated with IMD-0354 are shown as positive control (Figure 1D,I). Many inflammatory cells were found in the anterior and posterior segments at 24 h after LPS was administered. The eyes of naïve rats showed no inflammation (Figure 1E,J).

The infiltrating cells in the ICB area of the sections were counted and averaged. The mean numbers of the infiltrating cells were 6.0±1.0, 18.3±5.2, 23.3±2.8, and 51.7±5.6 in eyes treated with 30, 10, 3, and 0 mg/ml of IMD-0354, respectively. The numbers of infiltrating cells in the ICB area were significantly reduced in eyes treated with 30 mg/ml (p<0.01), 10 mg/ml (p<0.05), and 3 mg/ml (p<0.05) of IMD-0354 compared with those of vehicle alone in the EIU rats (Figure 1 K).

The infiltrating cells in the posterior area of the histological sections were also counted and averaged. The mean number of infiltrating cells was 64.0±2.1 in vehicle-given EIU eyes, and 9.0±1.5, 18.3±3.4, and 57.0±5.3 in eyes treated with 30, 10, and 3 mg/ml of IMD-0354, respectively. The number of infiltrating cells was significantly reduced in eyes treated with 30 or 10 mg/kg of IMD-0354 mg/ml compared to those of the positive controls (p<0.01, Figure 1L). The number of infiltrating cells decreased in a dose-dependent manner when treated with IMD-0354 in EIU.

### Effect of IMD-0354 on cellular infiltration and protein concentration in aqueous humor

**Figure 2:**
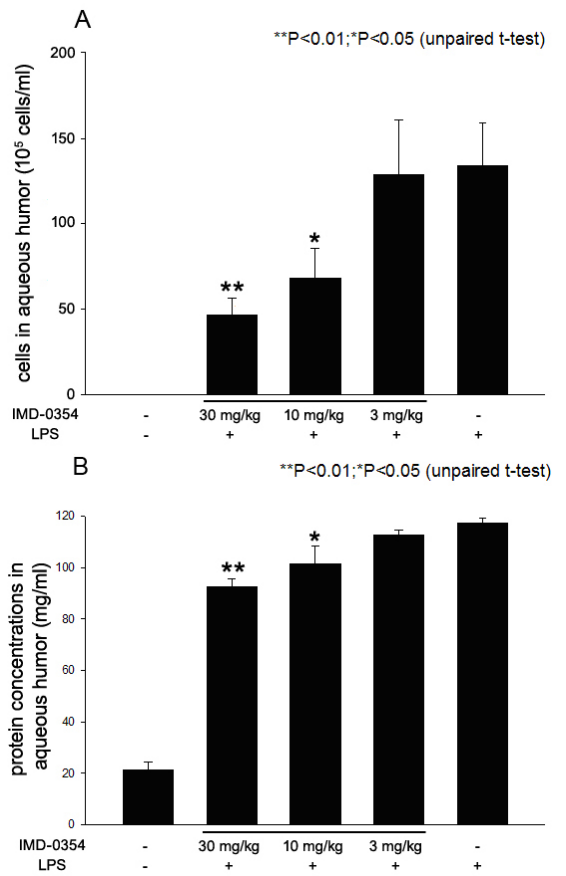
Effect of IMD-0354 on cellular infiltration **A**: and protein concentration **B**: in aqueous humor collected 24 h after lipopolysaccharide (LPS) treatment. Data are shown as mean±standard error of mean (SEM; n=8). *p<0.05, **p<0.01, significantly different from the LPS group.

Severe inflammation was found in the anterior segment of rats with EIU at 24 h after LPS was administered. In the LPS group, the number of inflammatory cells in aqueous humor 24 h after LPS was administered was 132.0±25.0×10^5^ cells/ml. The number of aqueous infiltrating cells was 53.6±9.8×10^5^, 72.5±17.0×10^5^, and 127.25±32.0×10^5^ cells/ml, when treated with 30, 10, and 3 mg/kg of IMD-0354, respectively. The mean number of aqueous inflammatory cells was significantly reduced by treatment with 30 mg/kg (p<0.01) and 10 mg/kg (p<0.05) of IMD-0354, whereas 3 mg/kg of IMD-0354 did not result in a significant reduction compared with the LPS group (Figure 2A). No infiltrating cells were detected in the aqueous humor of the naïve rats.

Next, we quantified the aqueous protein levels. A high level of protein was detected in the aqueous humor of untreated EIU rats as 117.33±1.8 mg/ml. The protein concentrations in rats treated with 30 mg/kg (92.6±3.1 mg/ml, p<0.01) and 10 mg/kg (101.5±6.8 mg/ml, p<0.05) of IMD-0354 were significantly lower than that in the LPS group, whereas 3 mg/kg of IMD-0354 did not result in a significant reduction in protein concentration (112.6±1.9 mg/ml) compared with the LPS group (Figure 2B). The decrease in the aqueous protein levels was shown as a dose-dependent manner of IMD-0354.

### Effect of IMD-0354 on tumor necrosis factor-α and monocyte chemoattractant protein-1 in aqueous humor

**Figure 3:**
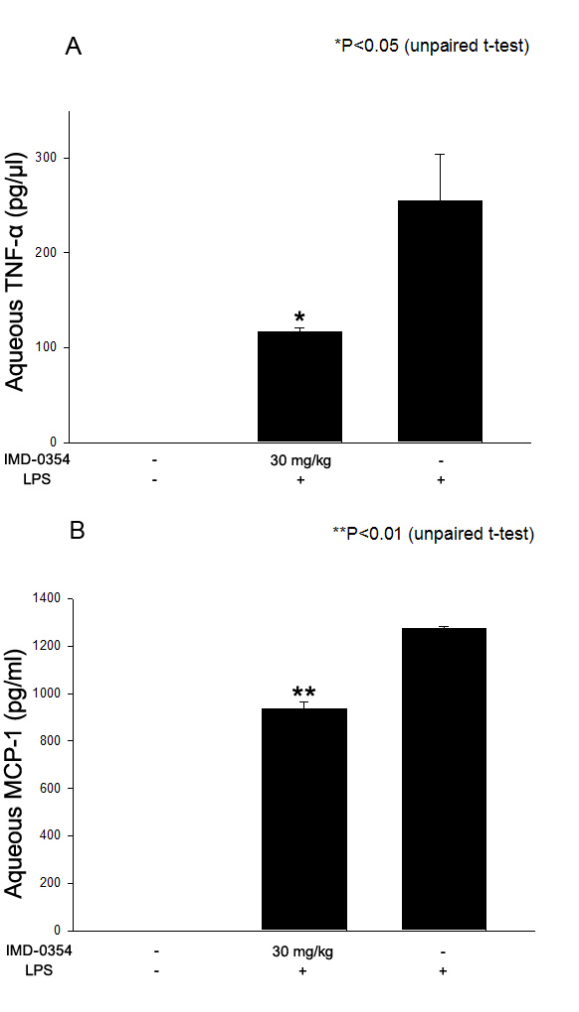
Effect of IMD-0354 (30 mg/kg) on tumor necrosis factor (TNF)-α **A**: and monocyte chemoattractant protein (MCP)-1 **B**: in aqueous humor collected 24 h after lipopolysaccharide (LPS) treatment. Data are shown as mean±standard error of mean (SEM; n=8). *p<0.05, **p<0.01, significantly different from the LPS group.

Considerable production of TNF-α and MCP-1 in the aqueous humor was seen in the untreated EIU rats. Treatment with 30 mg/kg of IMD-0354 significantly reduced TNF-α (p<0.05) and MCP-1 (p<0.01) concentrations in aqueous humor (Figure 3A,B). The TNF-α and MCP-1 levels in the aqueous humor of the naïve controls were below the sensitivity threshold of the appropriate ELISA kit.

### Immunohistochemistry of nuclear factor kappa B p65 translocation in iris-ciliary body

**Figure 4:**
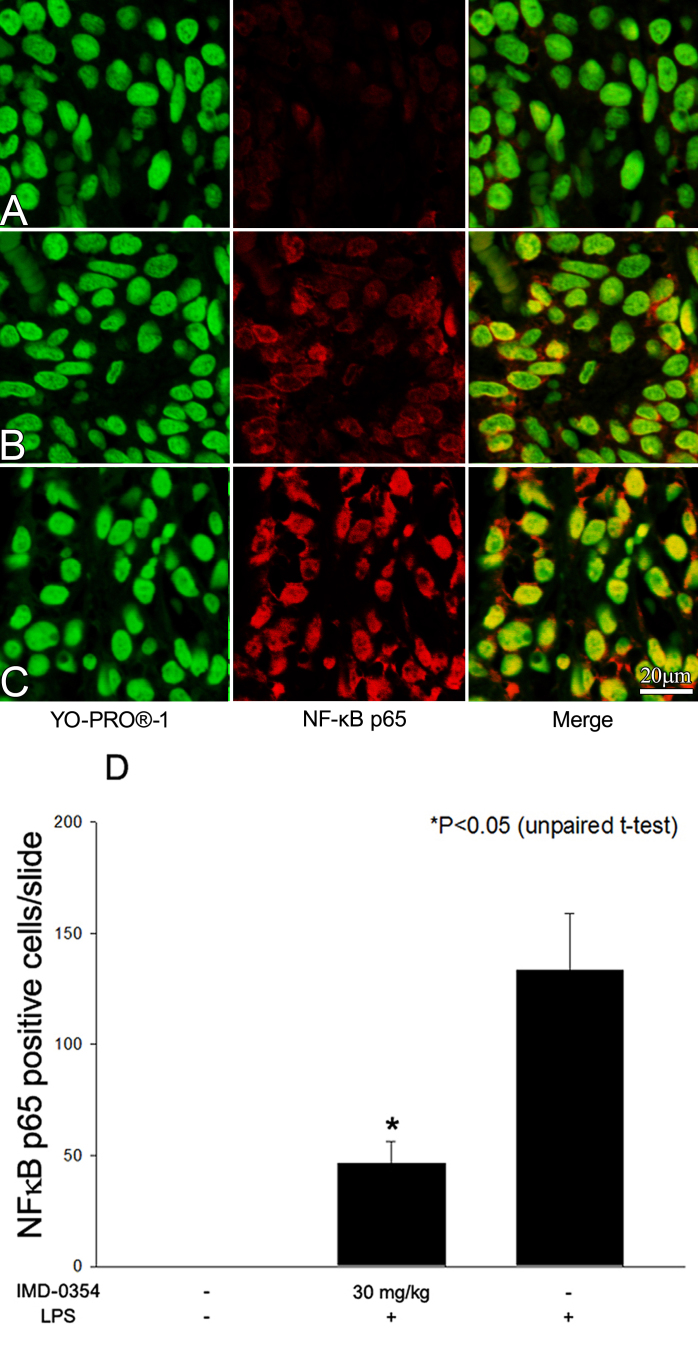
Effect of IMD-0354 on nuclear factor (NK) κB p65 (red) activation in the iris-ciliary body 3 h after lipopolysaccharide injection. Dual-immunofluorescence labeling showed the NFκB co-localization (yellow) in nuclei (green). Control animals **A**: were not injected with lipopolysaccharide (LPS); only weak NFκB signal detected in cytoplasm area of the cells, no nuclear co-localization of NF NFκB was detected. In the group of endotoxin-induced uveitis (EIU) rats treated with IMD-0354 30 mg/kg **B**: reductions of NFκB co-localization were observed compared to untreated EIU rats **C**: Quantitative analysis of NF-κB-positive cells in the iris-ciliary body (ICB) presented in graph **D**: Data are shown as mean±standard error of mean (n=4). *Significantly different from LPS group (p<0.05).

Three hours after LPS was injected, activated NFκB p65 immunoreactivity was examined. NFκB p65 was strongly expressed in the ICB of untreated EIU rats (Figure 4C). In contrast, the number of activated NFκB-positive cells was lower in the ICB of rats treated with IMD-0354 (Figure 4B), whereas the controls showed only background levels (Figure 4C). The active NFκB-positive nuclei were counted to obtain a quantitative measure of NFκB activity in the ICB (Figure 4D). LPS injection resulted in a significant increase in the number of active NFκB-positive cells in the ICB at 3 h (133.3±25.0). In the group treated with 30 mg/kg of IMD-0354, the number of active NFκB-positive cells was significantly decreased (46.3±9.8; p<0.05). In the naïve control group, no active NFκB-positive cells were detected in the ICB.

### Immunohistochemistry of phosphorylated I-κBα in iris-ciliary body

**Figure 5:**
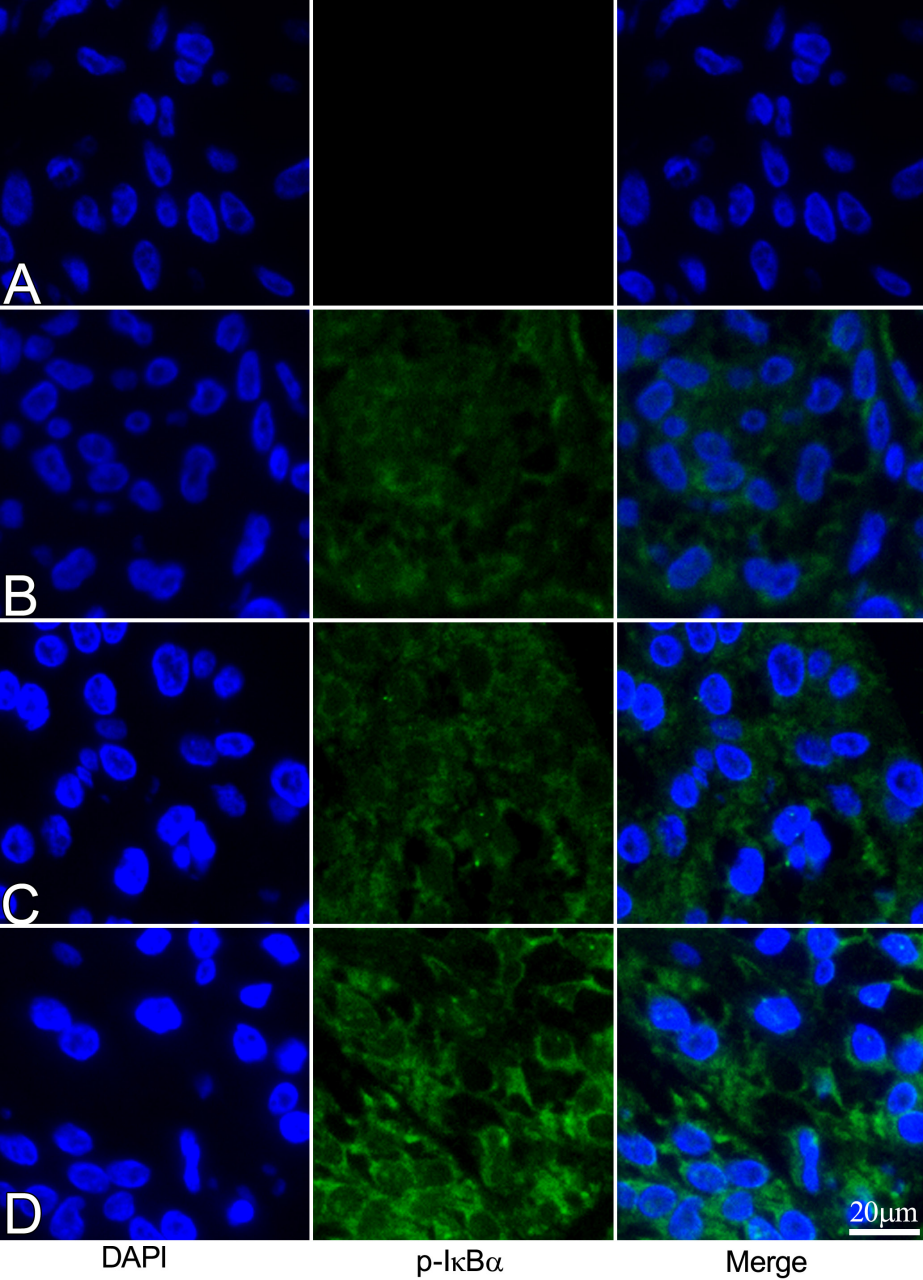
Effect of IMD-0354 on phosphorylated inhibitors of κB (green) in the iris-ciliary body 3 h after lipopolysaccharide injection. No phosphorylated inhibitor of κB (p-IκB signal was detected in negative control **A**: where no p-IκB antibodies were applied. The p-IκB signals were similarly expressed in cytoplasm of naïve controls **B**: and IMD-0354-treated (30 mg/kg) LPS injected rats **C**: Intensive p-IκB expression was observed in cytoplasm of iris-ciliary body (ICB) cells in untreated endotoxin-induced uveitis (EIU) rats **D**: Cell nuclei were stained with 4',6-diamidino-2-phenylindole (DAPI) as blue.

Three hours after LPS was injected, phosphorylated I-κBα immunoreactivity was examined. No phosphorylated I-κBα signal was detected in the negative controls (Figure 5A). Only background levels of phosphorylated I-κBα immunoreactivity was observed in the naïve (Figure 5B) or IMD-0354 treated EIU rats (Figure 5C). However, phosphorylated I-κBα was strongly expressed in the ICB cell cytoplasm of the IMD-0354-untreated EIU rats (Figure 5D).

### Effect of oral administration of IMD-1041, a prodrug of IMD-0354

**Figure 6:**
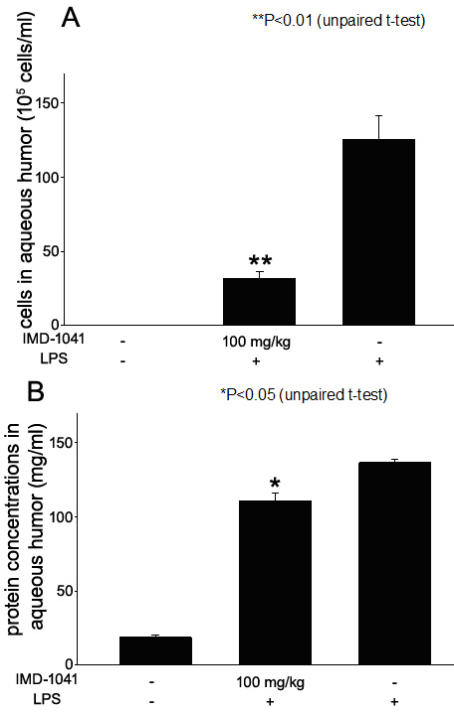
Effect of 100 mg/kg of IMD-1041 oral application on cellular infiltration **A**: and protein concentration **B**: in aqueous humor collected 24 h after lipopolysaccharide (LPS) treatment. Data are shown as mean±standard error of mean (SEM; n=4). *p<0.05, **p<0.01, significantly different from the LPS group.

To confirm the suppressive effects on EIU by IKKβ inhibitor IMD-0354, we examined the effects of its oral prodrug IMD-1041. The number of infiltrating cells in aqueous humor 24 h after LPS was injected was 125.7±16.0×10^5^ and 31.33±5.2×10^5^ cells/ml in rats untreated and treated with 100 mg/kg of IMD-1041, which significantly reduced the number of inflammatory cells (p<0.01, Figure 6A). No infiltrating cells were detected in the aqueous humor of naïve rats.

Aqueous protein levels were measured as 137.0±2.1 and 111.3±5.4 mg/ml in EIU rats untreated and treated with 100 mg/kg of IMD-1041. The aqueous protein level in the naïve rats was 18.6±1.7. The aqueous protein levels pretreated with IMD-1041 were significantly decreased compared with those of untreated EIU rats (p<0.05, Figure 6B).

## Discussion

We examined the therapeutic effects of IKKβ inhibitor IMD-0354 on EIU, an animal model for acute ocular inflammation. We observed a single administration of IMD-0354 significantly ameliorated EIU in rats. Aqueous cell infiltration and protein levels, inflammatory cytokines, and chemokines in the aqueous humor were also significantly decreased in EIU rats treated with IMD-0354 compared to those of the untreated EIU rats. NFκB is a key transcription factor that regulates various inflammatory processes [17]. In unstimulated cells, NFκB is found in cytoplasm and bound to IκBα and IκBβ. When cells are stimulated, NFκB is released from IκB by kinases phosphorylate IκB, and moves into the nucleus, where NFκB binds to specific sequences in the promoter regions of the target genes [18]. Proinflammatory cytokines and reactive oxygen species (ROS) activate NFκB in response to various stimuli, including LPS [17]. In the present study, NFκB p65 nuclear translocation in the ICB was significantly suppressed by a single administration of IMD-0354. Our immunohistochemical findings support that NFκB inhibition by IMD-0354 is achieved by preventing I-κBα phosphorylation, which corresponds to previous data [10,19]. Activating NFκB leads to an increase in the expression of several genes, including TNF-α and MCP-1, which mediate inflammation and immune responses. These cytokines sometimes activate, and on other occasions are activated by, NFκB [20].

MCP-1 is an important mediator of monocyte infiltration [21] and was shown to be overexpressed in human eyes during acute anterior uveitis [22], and during EIU in rats [2]. NFκB upregulates transcription of the MCP-1 gene [23] [24]. The elevated MCP-1 level was significantly decreased when treated with IMD-0354 in this study. This suggests that suppressing MCP-1 production by administering IMD-0354 leads to reduced monocyte recruitment in the inflamed ocular tissue. Thus, it seems that IMD-0354 inhibits the positive cycle of NFκB and TNF-α, which results in the anti-inflammatory effect in EIU rats. Furthermore, our present study demonstrated the suppressive effects on EIU of the prodrug form of IMD-0354, IMD-1041. NFκB inhibitors are promising agents for managing ocular inflammatory disorders. In addition, since IMD-0354 was shown to be more effective for aqueous cell numbers than protein concentrations, IKK inhibition may act on adhesion molecules of ICB in this model.

We previously showed several efficacious NF-κB inhibitors on experimental autoimmune uveoretinitis (EAU), an animal model for human endogenous uveitis [25,26]. However, they are injectable, and treatment with a high dose sometimes inflicts side effects in mice. The higher the specificity for IKK, the safer the NFκB inhibitors. Though pyrrolidine dithiocarbamate, a common NFκB inhibitor, shows cytotoxicity, no systemic and topical side effects due to IMD-0354/IMD-1041 administration were observed in this study.

In summary, inhibiting IκB phosphorylation to prevent nuclear translocation of NFκB attenuates inflammatory response in the eyes of rats. NFκB plays central and multiple roles in the immune response, as a mediator of many proinflammatory signal transductions, and therefore, an inhibitor of IKKβ may function as a multifaceted suppressor of inflammatory disorders in the eye. IMD-0354 and IMD-1041 seem to be promising candidate agents for ocular inflammatory disorders since they showed significant effects with no systemic side effects in this study; in addition, oral agents are less stressful for patients.
